# The relationship of miR-155 host gene polymorphism in the susceptibility of cancer: a systematic review and meta-analysis

**DOI:** 10.3389/fgene.2025.1517513

**Published:** 2025-03-06

**Authors:** Gang Jin, Tao Guo, Jia-Wei Liu, Han-Yu Yang, Jian-Guo Xu, Yao Pang, Yi Yang, Shao-E. He, Kang Yi

**Affiliations:** ^1^ Department of Thoracic Surgery, Gansu Provincial Hospital, Lanzhou, China; ^2^ Department of Cardiovascular Surgery, Gansu Provincial Hospital, Lanzhou, China; ^3^ The First School of Clinical Medicine of Gansu University of Chinese Medicine, Lanzhou, China; ^4^ Gansu International Scientific and Technological Cooperation Base of Diagnosis and Treatment of Congenital Heart Disease, Lanzhou, China; ^5^ Department of Thoracic Surgery, Qin’an County People’s Hospital, Tianshui, China; ^6^ Evidence-Based Medicine Center, School of Basic Medical Sciences, Lanzhou University, Lanzhou, China; ^7^ The First Hospital of Lanzhou University, Lanzhou, Gansu, China; ^8^ Department of Thoracic Surgery, Kangle County Lianlu Town Health Center, Linxia, Gansu, China

**Keywords:** cancer, MicroRNA-155, MIR155HG, single nucleotide polymorphism, meta-analysis

## Abstract

**Background:**

miR-155 is overexpressed in many cancers, highlighting its potential as a biomarker for cancer diagnosis, treatment, and therapeutic evaluation. miR-155 is processed from the miR-155 host gene (*MIR155HG*). Genetic variations in *MIR155HG* may influence cancer susceptibility, but existing evidence is inconclusive. This study aimed to evaluate the association of *MIR155HG* polymorphisms with cancer risk.

**Material/Methods:**

A systematic literature search identified 15 case-control studies on three single nucleotide polymorphisms (SNPs): rs767649 (T > A), rs928883 (G > A), and rs1893650 (T > C). Meta-analysis was performed using RevMan 5.4, with odds ratios (ORs) and 95% confidence intervals (CIs) as effect measures.

**Results:**

No significant association was observed for rs767649 and rs928883 in overall cancer analysis. However, subgroup analysis revealed rs767649 increased susceptibility to respiratory, digestive, and reproductive cancers, while reducing cancer risk after excluding reproductive cancers. rs928883 showed a protective effect for digestive cancers. rs1893650 was not significantly associated with cancer risk.

**Conclusion:**

*MIR155HG* polymorphisms influence susceptibility to specific cancer subtypes, particularly respiratory and digestive cancers. These findings underscore the importance of genetic and environmental factors in cancer risk and warrant further investigation.

## 1 Background

The onset of cancer originates from abnormalities in cell proliferation and apoptosis (Thompson, 1995), which result from dysregulation of intracellular signaling pathways governing these processes (Manzo-Merino et al., 2014). Cancer is a critical public health issue worldwide and remains one of the leading causes of morbidity and mortality globally (Siegel et al., 2023), and poses a significant global health burden. The etiology of cancer is highly complex, with factors such as smoking, alcohol consumption, environmental factors, and viral infections potentially contributing to the development of various types of cancer. Nevertheless, the exact mechanisms underlying cancer development are not yet fully understood. There is increasing evidence of a complex interplay between genetic and environmental factors in the initiation and progression of cancer (Lichtenstein et al., 2000; Pharoah et al., 2004). Among these, single nucleotide polymorphisms (SNPs) have been extensively studied as they may alter cancer susceptibility (Hanahan and Weinberg, 2011), indicating that the detection of genetic risk factors could suggest cancer risk and prognosis, further enhancing cancer diagnostic capabilities.

**FIGURE 1:**
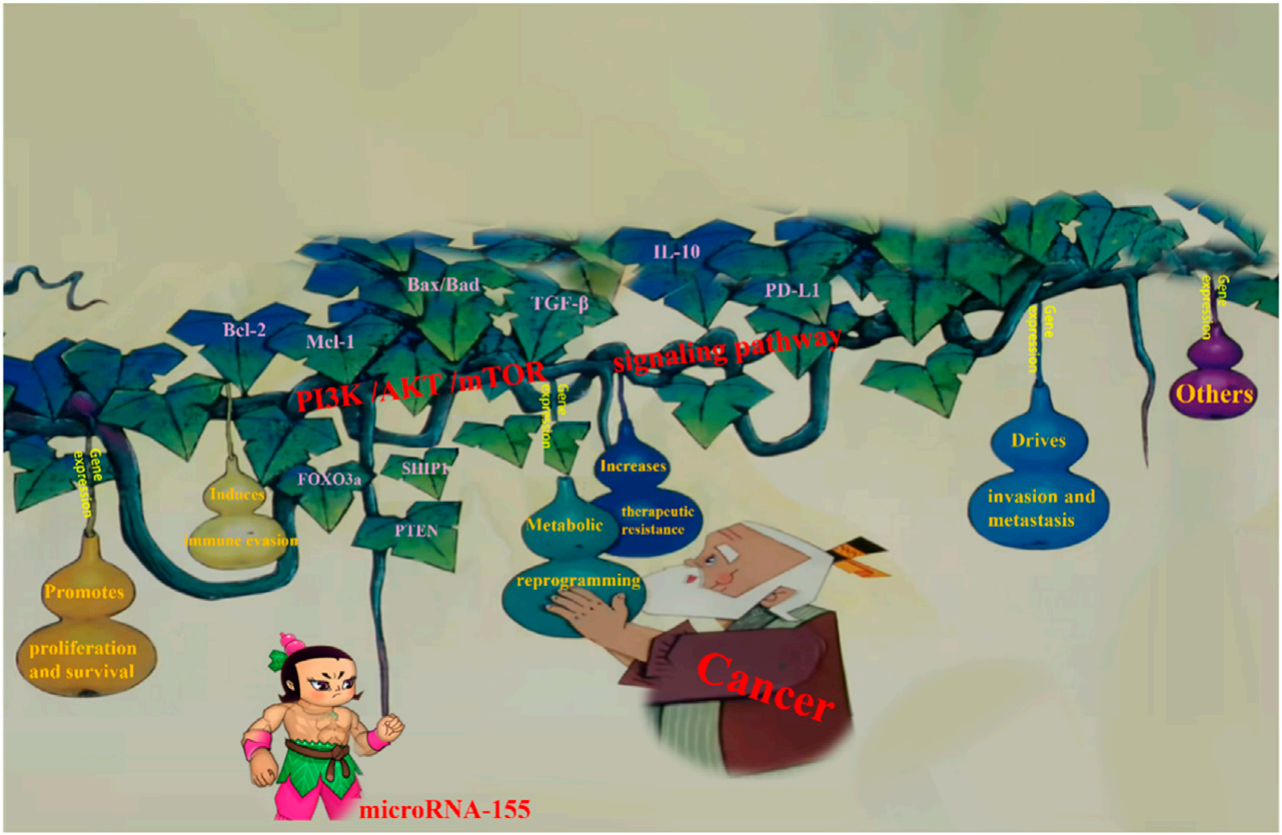
Molecular mechanisms of microRNA-155 in cancer progression via the PI3K/AKT/mTOR signaling pathway. microRNA-155 suppresses key negative regulators (SHIP1, PTEN), leading to the activation of the PI3K/AKT/mTOR signaling pathway. This activation drives tumor progression by promoting cell proliferation and survival through enhanced expression of anti-apoptotic molecules (Bcl-2, Mcl-1) and suppression of pro-apoptotic factors (Bax, Bad). It further facilitates invasion and metastasis via epithelial-mesenchymal transition, metabolic reprogramming, immune evasion, and therapy resistance.

microRNA (miRNA) refers to small, non-coding single-stranded RNA molecules encoded by endogenous genes, with a length of approximately 20–24 nucleotides. Mature miRNAs bind to the 3′untranslated region (3′UTR) of target gene mRNAs through sequence complementarity. When the miRNA and the target mRNA are not fully complementary, the expression of the target gene can be suppressed at the level of protein translation. In contrast, when the miRNA is fully or nearly fully in a complementary manner to the target mRNA, it leads to the degradation of the target gene (Bartel, 2004), thus regulating the expression of the target gene and mediating processes such as cell proliferation, metabolism, development, and differentiation. In 1993, Lee and colleagues first discovered that lin4 encoded miRNA in *C. elegans* (*Caenorhabditis elegans*) (Lee et al., 1993), which binds complementarily to the 3′UTR, controlling the development of *C. elegans* by inhibiting the expression of target gene mRNAs. microRNA-155 (miR-155) is located in the third exon of the non-coding gene B-cell integration cluster (BIC) on human chromosome 21. It is a non-coding RNA consisting of 23 nucleotides, and its expression is influenced by various steps of BIC transcription and miRNA processing. As a member of the miRNA family, miR-155 exhibits the biological functions typical for miRNAs, not only promote tumorigenesis by inducing mutational phenotypes that lead to a high mutation rate, but also create a gene expression environment that is especially susceptible to malignant transformation (Tili et al., 2011; Witten et al., 2019). miR-155 can directly or indirectly regulate the PI3K signaling pathway, inducing cell proliferation (Wu and Wang, 2020), migration (Fang et al., 2024), invasion, and apoptosis of tumor cells, thereby playing a role in the regulation of cancer development and progression (Kong et al., 2016; Wu et al., 2017) (Figure 1). A large body of research has demonstrated that miR-155 plays a role in various human cancers (Donnem et al., 2011; Sandhu et al., 2012), and circulating plasma levels of miR-155 can serve as a biomarker for predicting cancer risk (Lao and Le, 2019; Wu et al., 2023). Various clinical studies have shown that miR-155 is highly expressed in various malignant tumor tissues, including lung cancer, breast cancer, pancreatic cancer, gallbladder cancer, glioblastoma, and other solid tumors (Bera et al., 2014; Czubak et al., 2015; D'Urso et al., 2012; Erbes et al., 2015; Peng et al., 2015; Poltronieri et al., 2013), as well as in hematological malignancies such as acute myeloid leukemia, multiple myeloma, and B-cell lymphoma (Bi et al., 2015; Khalife et al., 2015; Sandhu et al., 2012). Some scholars have also suggested that miR-155 is under expressed in tumor cells, with low expression observed in esophageal cancer, melanoma, ovarian cancer, and others (Jia et al., 2023; Ling et al., 2013; Qin et al., 2013; Wang et al., 2016). miR-155 is processed from *MIR155HG*, and genetic variations in *MIR155HG* can affect the expression of miR-155 (Wu et al., 2017). Currently, most of the research on the polymorphism of *MIR155HG* focuses on three sites: rs767649 (T > A), located in the promoter region of −1,570 bp upstream of *MIR155HG*, is the most studied SNP for *MIR155HG*; rs928883 (G > A) located in 2.3 kb upstream of *MIR155HG* in the second intron of *MIR155HG* (Schuetz et al., 2012); rs1893650 (T > C) located in intron 1 of *MIR155HG*, approximately 1.5 kb upstream of the transcription start site (TSS) of the gene. This positioning plays a crucial role in the regulation biological functions of miR-155, particularly its impact on cancer-related pathways such as the PI3K/Akt signaling pathway (Karajovic et al., 2024; Wu W. et al., 2019). The study reports on the correlation between three SNPs in *MIR155HG* and cancer (Ji et al., 2016; Schuetz et al., 2012; Wang et al., 2016; Wu H. et al., 2019; Xie et al., 2015; Zou et al., 2020). However, different studies have drawn inconsistent conclusions (Dezfuli et al., 2020; Ji et al., 2016; Xie et al., 2015), and the association between these SNPs and cancer development remains unclear.

The current research has reported the association between three SNPs of *MIR155HG* and cancer (Dezfuli et al., 2020; Iranparast et al., 2023; Ji et al., 2016; Karajovic et al., 2024; Wu H. et al., 2019; Wu et al., 2017; Xie et al., 2015; Zou et al., 2020), though conclusions from different studies are inconsistent. In addition, the relationship between *MIR155HG* polymorphisms and overall human cancer risk has not yet been comprehensively analyzed. To further investigate this issue, this study is the first to utilize a systematic review based on meta-analysis to explore the association between *MIR155HG* polymorphisms and cancer. It also examines the differences among subgroups, including respiratory system-related cancers, digestive system-related cancers, reproductive system-related cancers, as well as cancers in different regions and ethnic groups.

## 2 Materials and methods

### 2.1 Literature search

**FIGURE 2:**
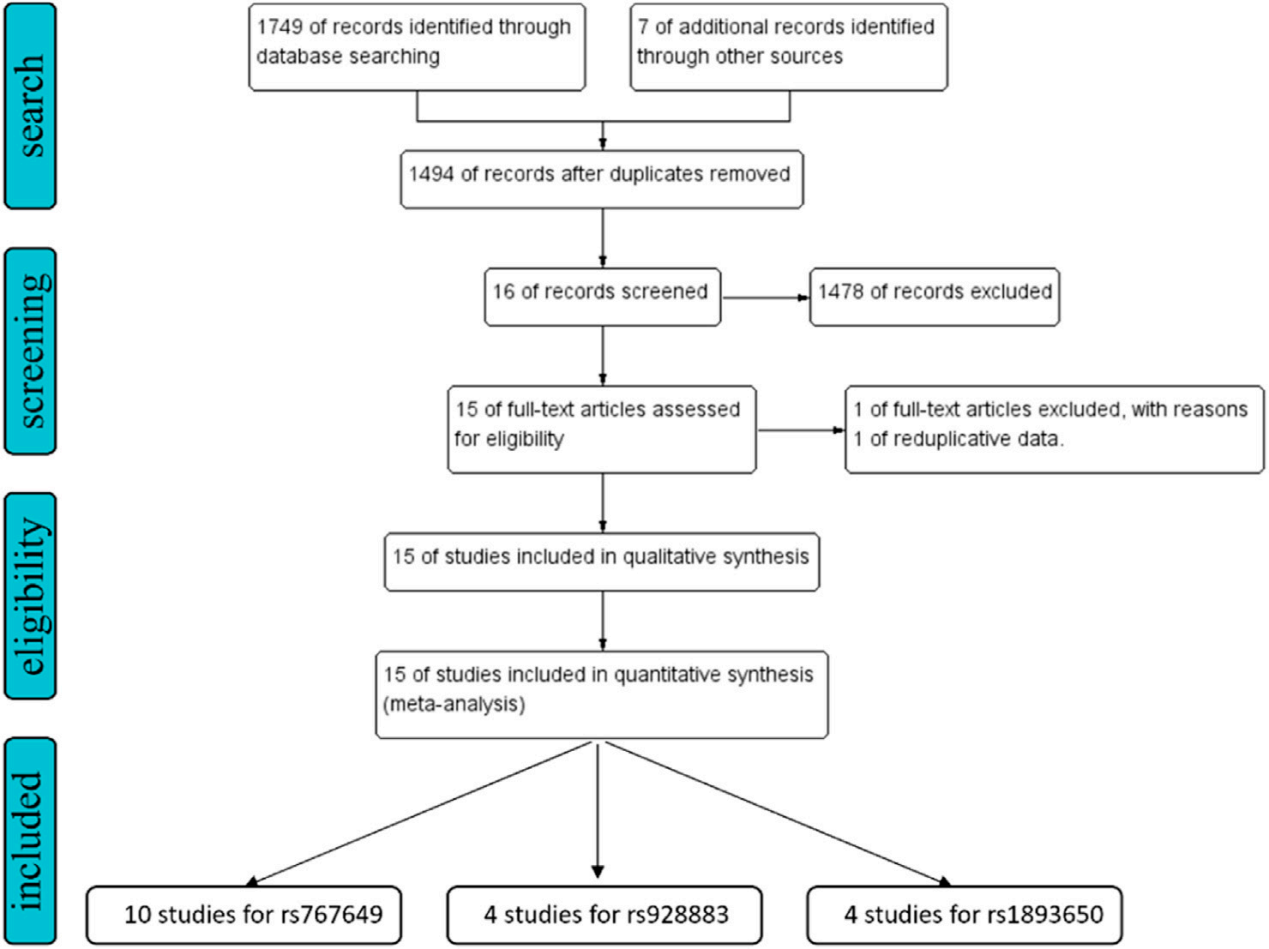
The flow chart of study selection for the present study. We expanded the search scope to “related articles.”

A systematic literature study was conducted on 7 databases including PubMed, Embase, Web of Science, Cochrane Library, China National Knowledge Infrastructure, VIP, Wan Fang to retrieve all relevant articles before 10 October 2024. All retrieval studies are manually searched and selected (Figure 2). The retrieval process is described in the supplementary document (Supplementary Material).

### 2.2 Inclusion and exclusion criteria

The inclusion criteria for this study were determined before the literature search. The included studies needed to meet the following criteria: (1) association studies between *MIR155HG* polymorphisms and cancer; (2) case-control studies; (3) detailed genotype data can be obtained by calculated odds ratios (OR) and 95% confidence intervals (CIs); (4) distribution of genotypes in the control group is consistent with Hardy-Weinberg equilibrium (HWE); (5) Include original research written in either English or Chinese.

Exclusion criteria: (1) reviews, letters, comments, expert opinions, case reports, and family-based association studies; (2) repetition of previous publications; (3) animal-based studies or cell line research.

### 2.3 Data extraction and risk of bias

The following data were independently extracted according to inclusion and exclusion criteria: the first author, publication year, country and region of study, genotyping method, type of cancers, source of control population, case and control sample size, genotype frequencies of *MIR155HG* polymorphisms in case and control, and results of the HWE test (Table 1). The risk of bias in the included literature was referenced to the Newcastle-Ottawa scale scoring standard (Table 3). The scoring system evaluated the included studies from 3 aspects: (1) the selectivity of the case and the control group; (2) the comparability of the case and the control group; (3) the exposure of the risk factors. The highest score achievable is 9 points. It is widely accepted that a research study was considered high-quality when the score was ≥7, and it is considered to be a study with low risk of bias.

**TABLE 1:** Characteristics of included studies.

Gentic	First author	Year	Country	Region	Genotyping method	Case type	Controls source	PHWE
rs767649	Karajovic, J.	2024	Serbia	Europe	PCR	PTC	HB	0.57
	Iranparast, S.	2023	Iran	Asian	PCR-RFLP	BC	HB	0.03
	Yaheng Li	2021	China	Asian	TaqMan Assay	CC	HB	0.17
	Dezfuli, Neda K.	2021	Iran	Asian	PCR-RFLP	NSCLC	HB	0.15
	Dezfuli, N. K.	2020	Iran	Asian	PCR-RFLP	NSCLC	HB	0.17
	Xiaona Wang	2018	China	Asian	TaqMan Assay	CC	HB	0.91
	Dingguo Pan	2018	China	Asian	TaqMan Assay	CRC	HB	0.61
	Shizhi Wang	2016	China	Asian	TaqMan SNP genotyping Assay	CC	HB	0.09
	Jiansong Ji	2016	China	Asian	Sequenom MassARRAY	HCC	HB	0.6
	Kaipeng Xie	2015	China	Asian	Sequenom MassARRAY	NSCLC	HB	0.84
rs928883	Zhuoqi Jia	2023	China	Asian	Agena MassARRAY	ESCC	HB	0.3
	Xu Chao	2020	China	Asian	Agena MassARRAY	HCC	HB	0.58
	Huangfu Wu	2019	China	Asian	Agena MassARRAY	CRC	HB	0.92
	Schuetz, J. M.	2012	Canada	North America	Sanger	NHL	HB	0.05
rs1893650	Karajovic, J.	2024	Serbia	Europe	PCR	PTC	HB	0.48
	Wenjing Zou	2020	China	Asian	MassARRAY	GC	HB	0.56
	Xu Chao	2020	China	Asian	Agena MassARRAY	HCC	HB	0.9
	Huangfu Wu	2019	China	Asian	Agena MassARRAY	CRC	HB	0.79

PTC, papillary thyroid cancer; BC, breast cancer; CC, cervical cancer; NSCLC, non-small cell lung; CRC, colorectal cancer; ESCC, esophageal cancer; GC, Gastric cancer; NHL, non-Hodgkin lymphoma; HCC, Hepatocellular carcinoma. HWE, hardy weinberge quilibrium; HB, hospital-based.

### 2.4 Statistical analysis

All data analysis was performed using RevMan5.4 software. The OR and 95% CIs were calculated among 5 genetic models including allele model, homozygous model, heterozygous model, dominant model, and recessive model. We performed heterogeneity tests on the included studies using the Q test and I^2^ test. The fixed-effects model was only used for analysis when P > 0.10 and I^2^ ≤ 50%. Otherwise, the heterogeneity of this study was considered significant, and the random-effects model was used for analysis. We considered the analysis results to be significant when P value <0.05.

## 3 Results

### 3.1 Characteristics of included studies

The literature search identified 1756 articles, and based on the inclusion and exclusion criteria, 15 studies were included in the final analysis, including 12 English articles (Chao et al., 2020; Dezfuli et al., 2020; Dezfuli et al., 2021; Iranparast et al., 2023; Ji et al., 2016; Jia et al., 2023; Karajovic et al., 2024; Wang et al., 2016; Wu H. et al., 2019; Xie et al., 2015; Zou et al., 2020) and 3 Chinese articles (Supplementary Material). After organizing the data, rs767649 (T > A), rs928883 (G > A), and rs1893650 (T > C) were included in5309 cases and6304 controls, 2176cases and 1957 controls, and 1,554 cases and 1,547 controls, respectively. The characteristics of all selected articles are summarized in Table 1, while Table 2 examines the genotype features of the included studies. Additionally, Table 3 presents the bias risks associated with the results obtained from the 15 included studies.

**TABLE 2:** Genotype characteristics of included studies (rs767649).

Case type	First author									Allele frequencies		Allele frequencies	
		Cases				Controls				Cases		Controls	
		Total	TT	TA	AA	Total	TT	TA	AA	T	A	T	A
Cancer	Karajovic, J.	102	86	16	0	106	95	11	0	0.92	0.08	0.95	0.05
	Iranparast, S.	174	132	35	7	129	93	29	7	0.86	0.14	0.83	0.17
	Yaheng Li	438	153	211	74	511	180	259	72	0.59	0.41	0.6	0.4
	Dezfuli, Neda K.	33	19	10	4	30	17	9	4	0.73	0.27	0.71	0.28
	Dezfuli, N. K.	165	131	28	6	147	102	38	7	0.88	0.12	0.82	0.18
	Xiaona Wang	245	100	107	38	416	149	199	68	0.63	0.37	0.6	0.4
	Dingguo Pan	154	34	70	50	203	26	98	79	0.45	0.55	0.37	0.63
	Shizhi Wang	1,157	119	585	453	1,280	188	642	450	0.36	0.64	0.4	0.6
	Jiansong Ji	1,500	277	735	488	1,500	221	697	582	0.43	0.57	0.38	0.62
	Kaipeng Xie	1,341	225	631	485	1982	276	933	773	0.4	0.6	0.37	0.63
Respiratory system cancers	Dezfuli, Neda K.	33	19	10	4	30	17	9	4	0.73	0.27	0.71	0.28
	Dezfuli, N. K.	165	131	28	6	147	102	38	7	0.88	0.12	0.82	0.18
	Kaipeng Xie	1,341	225	631	485	1982	276	933	773	0.4	0.6	0.37	0.63
Digestive system cancers	Dingguo Pan	154	34	70	50	203	26	98	79	0.45	0.55	0.37	0.63
	Jiansong Ji	1,500	277	735	488	1,500	221	697	582	0.43	0.57	0.38	0.62
Reproductive system cancers	Iranparast, S.	174	132	35	7	129	93	29	7	0.86	0.14	0.83	0.17
	Yaheng Li	438	153	211	74	511	180	259	72	0.59	0.41	0.6	0.4
	Xiaona Wang	245	100	107	38	416	149	199	68	0.63	0.37	0.6	0.4
	Shizhi Wang	1,157	119	585	453	1,280	188	642	450	0.36	0.64	0.4	0.6
Cancer	Karajovic, J.	102	86	16	0	106	95	11	0	0.92	0.08	0.95	0.05
(Exclusion of reproductive system cancers)	Dezfuli, Neda K.	33	19	10	4	30	17	9	4	0.73	0.27	0.71	0.28
	Dezfuli, N. K.	165	131	28	6	147	102	38	7	0.88	0.12	0.82	0.18
	Dingguo Pan	154	34	70	50	203	26	98	79	0.45	0.55	0.37	0.63
	Jiansong Ji	1,500	277	735	488	1,500	221	697	582	0.43	0.57	0.38	0.62
	Kaipeng Xie	1,341	225	631	485	1982	276	933	773	0.4	0.6	0.37	0.63
Asian	Iranparast, S.	174	132	35	7	129	93	29	7	0.86	0.14	0.83	0.17
	Yaheng Li	438	153	211	74	511	180	259	72	0.59	0.41	0.6	0.4
	Dezfuli, Neda K.	33	19	10	4	30	17	9	4	0.73	0.27	0.71	0.28
	Dezfuli, N. K.	165	131	28	6	147	102	38	7	0.88	0.12	0.82	0.18
	Xiaona Wang	245	100	107	38	416	149	199	68	0.63	0.37	0.6	0.4
	Dingguo Pan	154	34	70	50	203	26	98	79	0.45	0.55	0.37	0.63
	Shizhi Wang	1,157	119	585	453	1,280	188	642	450	0.36	0.64	0.4	0.6
	Jiansong Ji	1,500	277	735	488	1,500	221	697	582	0.43	0.57	0.38	0.62
	Kaipeng Xie	1,341	225	631	485	1982	276	933	773	0.4	0.6	0.37	0.63
Europe	Karajovic, J.	102	86	16	0	106	95	11	0	0.92	0.08	0.95	0.05
East Asian	Yaheng Li	438	153	211	74	511	180	259	72	0.59	0.41	0.6	0.4
	Xiaona Wang	245	100	107	38	416	149	199	68	0.63	0.37	0.6	0.4
	Dingguo Pan	154	34	70	50	203	26	98	79	0.45	0.55	0.37	0.63
	Shizhi Wang	1,157	119	585	453	1,280	188	642	450	0.36	0.64	0.4	0.6
	Jiansong Ji	1,500	277	735	488	1,500	221	697	582	0.43	0.57	0.38	0.62
	Kaipeng Xie	1,341	225	631	485	1982	276	933	773	0.4	0.6	0.37	0.63
Caucasian	Karajovic, J.	102	86	16	0	106	95	11	0	0.92	0.08	0.95	0.05
	Iranparast, S.	174	132	35	7	129	93	29	7	0.86	0.14	0.83	0.17
	Dezfuli, Neda K.	33	19	10	4	30	17	9	4	0.73	0.27	0.71	0.28
	Dezfuli, N. K.	165	131	28	6	147	102	38	7	0.88	0.12	0.82	0.18

Genotype characteristics of included studies (rs928883).													
Case type	First author									Allele frequencies		Allele frequencies	
		Cases				Controls				Cases		Controls	
		Total	GG	GA	AA	Total	GG	GA	AA	G	A	G	A
Cancer	Zhuoqi Jia	511	126	272	113	479	100	250	129	0.51	0.49	0.47	0.53
	Xu Chao	419	90	244	85	423	129	204	90	0.51	0.49	0.55	0.45
	Huangfu Wu	505	175	242	88	509	148	254	107	0.59	0.41	0.54	0.46
	Schuetz, J. M.	741	559	158	24	546	430	114	2	0.86	0.14	0.89	0.11
Carcinoma	Zhuoqi Jia	511	126	272	113	479	100	250	129	0.51	0.49	0.47	0.53
	Xu Chao	419	90	244	85	423	129	204	90	0.51	0.49	0.55	0.45
	Huangfu Wu	505	175	242	88	509	148	254	107	0.59	0.41	0.54	0.46
Digestive system cancers	Zhuoqi Jia	511	126	272	113	479	100	250	129	0.51	0.49	0.47	0.53
	Xu Chao	419	90	244	85	423	129	204	90	0.51	0.49	0.55	0.45
	Huangfu Wu	505	175	242	88	509	148	254	107	0.59	0.41	0.54	0.46
Asian	Zhuoqi Jia	511	126	272	113	479	100	250	129	0.51	0.49	0.47	0.53
	Xu Chao	419	90	244	85	423	129	204	90	0.51	0.49	0.55	0.45
	Huangfu Wu	505	175	242	88	509	148	254	107	0.59	0.41	0.54	0.46
North America	Schuetz, J. M.	741	559	158	24	546	430	114	2	0.86	0.14	0.89	0.11
East Asian	Zhuoqi Jia	511	126	272	113	479	100	250	129	0.51	0.49	0.47	0.53
	Xu Chao	419	90	244	85	423	129	204	90	0.51	0.49	0.55	0.45
	Huangfu Wu	505	175	242	88	509	148	254	107	0.59	0.41	0.54	0.46
Caucasian	Schuetz, J. M.	741	559	158	24	546	430	114	2	0.86	0.14	0.89	0.11

Genotype characteristics of included studies (rs1893650).													
Case type	First author									Allele frequencies		Allele frequencies	
		Cases				Controls				Cases		Controls	
		Total	TT	TC	CC	Total	TT	TC	CC	T	C	T	C
Cancer	Karajovic, J	102	75	17	10	106	53	46	7	0.82	0.18	0.72	0.28
	Wenjing Zou	506	350	121	35	500	343	140	17	0.81	0.19	0.83	0.17
	Xu Chao	432	281	145	6	431	279	135	17	0.82	0.18	0.8	0.2
	Huangfu Wu	514	326	165	23	510	355	140	15	0.8	0.2	0.83	0.17
Digestive system cancers	Wenjing Zou	506	350	121	35	500	343	140	17	0.81	0.19	0.83	0.17
	Xu Chao	432	281	145	6	431	279	135	17	0.82	0.18	0.8	0.2
	Huangfu Wu	514	326	165	23	510	355	140	15	0.8	0.2	0.83	0.17
Asian	Wenjing Zou	506	350	121	35	500	343	140	17	0.81	0.19	0.83	0.17
	Xu Chao	432	281	145	6	431	279	135	17	0.82	0.18	0.8	0.2
	Huangfu Wu	514	326	165	23	510	355	140	15	0.8	0.2	0.83	0.17
Europe	Karajovic, J.	102	75	17	10	106	53	46	7	0.82	0.18	0.72	0.28
East Asian	Wenjing Zou	506	350	121	35	500	343	140	17	0.81	0.19	0.83	0.17
	Xu Chao	432	281	145	6	431	279	135	17	0.82	0.18	0.8	0.2
	Huangfu Wu	514	326	165	23	510	355	140	15	0.8	0.2	0.83	0.17
Caucasian	Karajovic, J.	102	75	17	10	106	53	46	7	0.82	0.18	0.72	0.28

**TABLE 3:** Results of Newcastle-Ottawa scale quality evaluation included in the study.

Inclusion study	Study population selection				Group-to-group	Comparison of exposure factors			Total
	1)	2)	3)	4)	5)	6)	7)	8)	(minutes)
Karajovic, J.	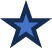	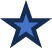	—	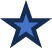	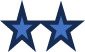	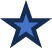	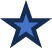	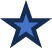	8
Zhuoqi Jia	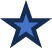	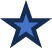	—	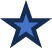	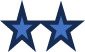	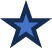	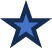	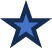	8
Iranparast, S.	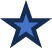	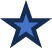	—	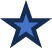	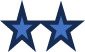	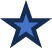	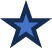	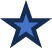	8
Yaheng Li	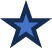	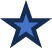	—	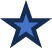	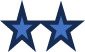	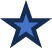	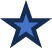	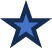	8
Dezfuli, Neda K.	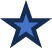	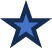	—	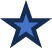	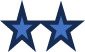	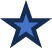	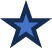	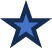	8
Wenjing Zou	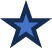	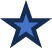	—	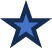	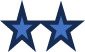	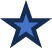	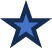	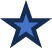	8
Xu Chao	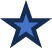	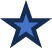	—	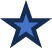	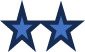	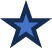	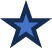	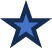	8
Dezfuli, N. K.	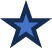	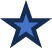	—	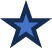	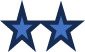	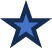	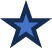	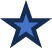	8
Huangfu Wu	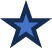	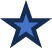	—	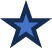	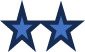	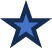	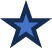	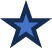	8
Xiaona Wang	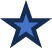	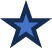	—	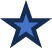	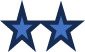	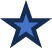	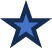	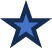	8
Dingguo Pan	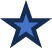	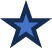	—	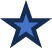	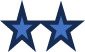	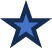	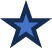	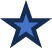	8
Shizhi Wang	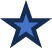	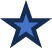	—	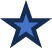	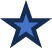	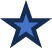	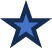	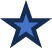	7
Jiansong Ji	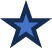	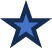	—	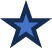	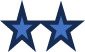	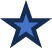	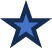	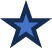	8
Kaipeng Xie	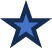	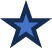	—	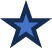	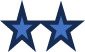	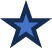	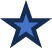	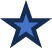	8
Schuetz, J. M.	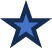	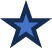	—	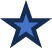	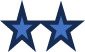	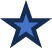	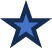	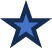	8

### 3.2 The analysis of *MIR155HG* rs767649 (T > A) polymorphism

#### 3.2.1 The analysis of *MIR155HG* rs767649 (T > A) polymorphism in overall cancer

**FIGURE 3:**
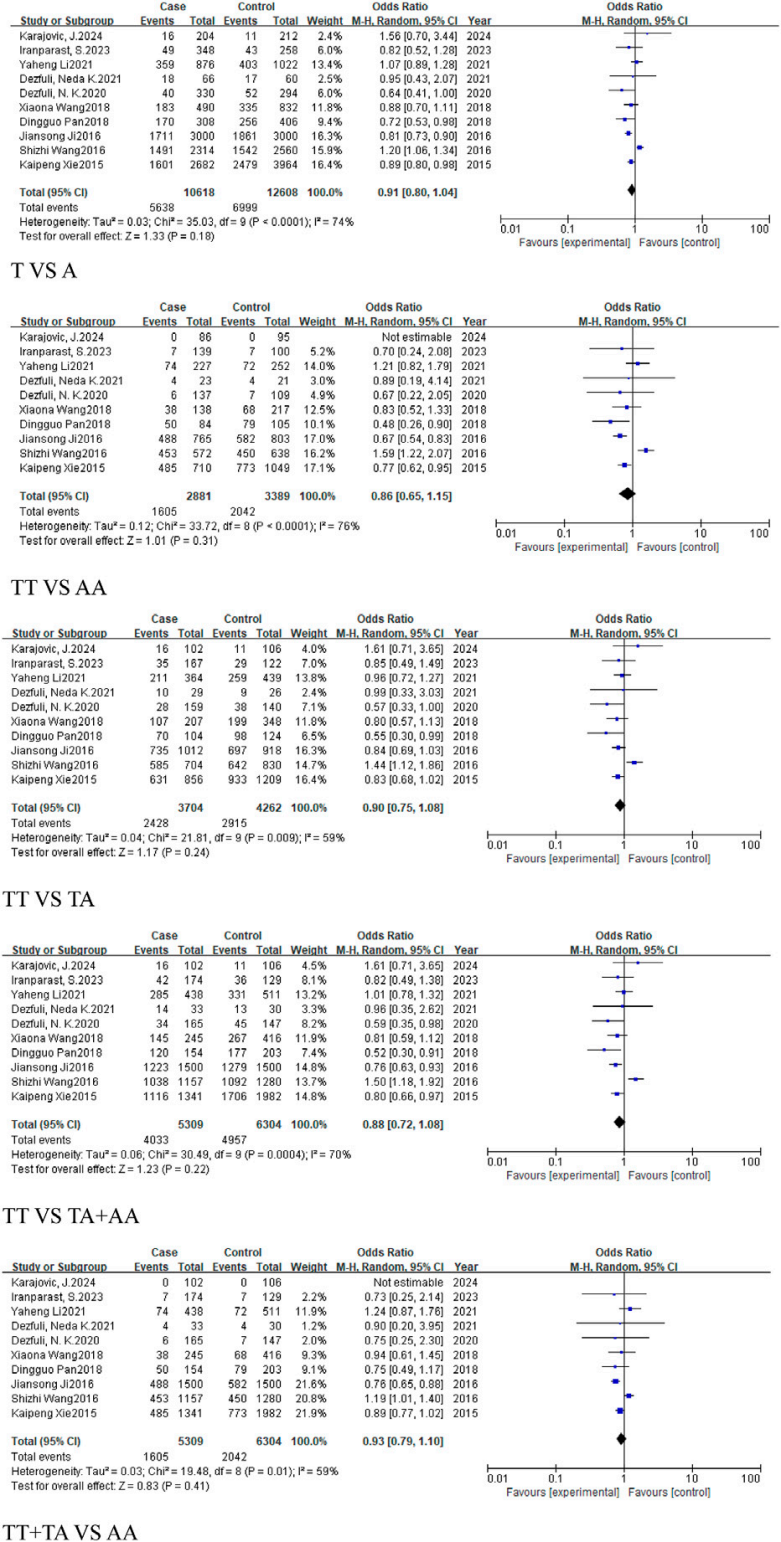
Forest plot of correlation between the microRNA-155 rs767649 (T > A) polymorphism and Cancer.

This analysis aimed to assess the association between the rs767649 (T > A) polymorphism and cancer risk. Ten studies were included, comprising 5,309 cases and 6,304 controls. Due to high heterogeneity, a random-effects model was applied (Table 4). The results showed no statistically significant association across all genetic models: allelic model (T vs. A, OR = 0.91, 95% CI [0.80, 1.04]), homozygous model (TT vs. AA, OR = 0.86, 95% CI [0.65, 1.15]), heterozygous model (TT vs. TA, OR = 0.9, 95% CI [0.75, 1.08]), dominant model (TA + AA vs. TT, OR = 0.88, 95% CI [0.72, 1.08]), and recessive model (TT + TA vs. AA, OR = 0.93, 95% CI [0.79, 1.10]). These results suggested that no significant association was found between the rs767649 polymorphism and cancer risk (P > 0.05) (Figure 3), and further studies are needed to confirm its role in cancer susceptibility.

**TABLE 4:** The meta-analysis results of microRNA-155 rs767649 (T > A) polymorphism and its correlation with the risk of cancer and its subgroups.

	Type	OR (95%)	95% CI	z	p	Test of heterogeneity		Analysis model
						I2	P*	
Cancer (10)	T vs. A	0.91	[0.80, 1.04]	1.33	0.18	74	<0.0001	Random-effects model
	TT vs. AA	0.86	[0.65, 1.15]	1.01	0.31	76	<0.0001	Random-effects model
	TT vs. TA	0.9	[0.75, 1.08]	1.17	0.24	59	0.009	Random-effects model
	TA + AA vs. TT	0.88	[0.72, 1.08]	1.23	0.22	70	0.0004	Random-effects model
	TT + TA vs. AA	0.93	[0.79, 1.10]	0.83	0.41	59	0.01	Random-effects model
Respiratory system cancers (3)	T vs. A	0.87	[0.79, 0.96]	2.71	0.007	0	0.37	Fixed-effects model
	TT vs. AA	0.77	[0.63, 0.94]	2.53	0.01	0	0.95	Fixed-effects model
	TT vs. TA	0.8	[0.66, 0.96]	2.35	0.02	0	0.44	Fixed-effects model
	TA + AA vs. TT	0.78	[0.65, 0.93]	2.79	0.005	0	0.49	Fixed-effects model
	TT + TA vs. AA	0.88	[0.77, 1.02]	1.71	0.09	0	0.96	Fixed-effects model
Digestive system cancers (2)	T vs. A	0.8	[0.73, 0.88]	4.42	<0.0001	0	0.47	Fixed-effects model
	TT vs. AA	0.65	[0.53, 0.79]	4.23	<0.0001	0	0.33	Fixed-effects model
	TT vs. TA	0.8	[0.66, 0.98]	2.21	0.03	45	0.18	Fixed-effects model
	TA + AA vs. TT	0.73	[0.61, 0.88]	3.34	0.0008	39	0.2	Fixed-effects model
	TT + TA vs. AA	0.76	[0.66, 0.88]	3.79	0.0001	0	0.97	Fixed-effects model
Reproductive system cancers (4)	T vs. A	1.04	[0.88, 1.22]	0.43	0.67	59	0.06	Random-effects model
	TT vs. AA	1.16	[0.82, 1.64]	0.86	0.39	57	0.07	Random-effects model
	TT vs. TA	1.02	[0.76, 1.36]	0.1	0.92	67	0.03	Random-effects model
	TA + AA vs. TT	1.03	[0.76, 1.41]	0.21	0.84	73	0.01	Random-effects model
	TT + TA vs. AA	1.16	[1.01, 1.33]	2.04	0.04	0	0.61	Fixed-effects model
Cancer (Exclusion of reproductive system cancers) (6)	T vs. A	0.84	[0.79, 0.90]	4.92	<0.00001	21	0.28	Fixed-effects model
	TT vs. AA	0.7	[0.61, 0.81]	4.8	<0.00001	0	0.66	Fixed-effects model
	TT vs. TA	0.82	[0.71, 0.93]	2.99	0.003	19	0.29	Fixed-effects model
	TA + AA vs. TT	0.77	[0.68, 0.87]	4.1	<0.0001	22	0.27	Fixed-effects model
	TT + TA vs. AA	0.82	[0.74, 0.91]	3.89	0.0001	0	0.69	Fixed-effects model
Asian (9)	T vs. A	0.9	[0.79, 1.03]	1.51	0.13	76	<0.0001	Random-effects model
	TT vs. AA	0.86	[0.65, 1.15]	1.01	0.31	76	<0.0001	Random-effects model
	TT vs. TA	0.88	[0.73, 1.05]	1.41	0.16	60	0.01	Random-effects model
	TA + AA vs. TT	0.86	[0.70, 1.05]	1.48	0.14	72	0.0004	Random-effects model
	TT + TA vs. AA	0.93	[0.79, 1.10]	0.83	0.41	59	0.01	Random-effects model
East Asian (6)	T vs. A	0.93	[0.80, 1.08]	0.98	0.33	84	<0.0001	Random-effects model
	TT vs. AA	0.88	[0.64, 1.23]	0.75	0.45	85	<0.00001	Random-effects model
	TT vs. TA	0.91	[0.73, 1.12]	0.91	0.36	71	0.004	Random-effects model
	TA + AA vs. TT	0.89	[0.69, 1.13]	0.96	0.34	81	<0.0001	Random-effects model
	TT + TA vs. AA	0.94	[0.79, 1.13]	0.62	0.53	74	0.002	Random-effects model
Caucasian (4)	T vs. A	0.82	[0.63, 1.08]	1.4	0.16	21	0.28	Fixed-effects model
	TT vs. AA	0.72	[0.36, 1.45]	0.91	0.36	0	0.95	Fixed-effects model
	TT vs. TA	0.83	[0.59, 1.16]	1.08	0.28	30	0.23	Fixed-effects model
	TA + AA vs. TT	0.82	[0.60, 1.12]	1.28	0.2	30	0.23	Fixed-effects model
	TT + TA vs. AA	0.77	[0.39, 1.53]	0.74	0.46	0	0.97	Fixed-effects model

#### 3.2.2 The analysis of *MIR155HG* rs767649 (T > A) polymorphism in cancer subgroups

This analysis examined the association between the rs767649 (T > A) polymorphism and cancer risk in respiratory, digestive, reproductive system-related cancers, and cancers excluding reproductive system cancers.

**FIGURE 4:**
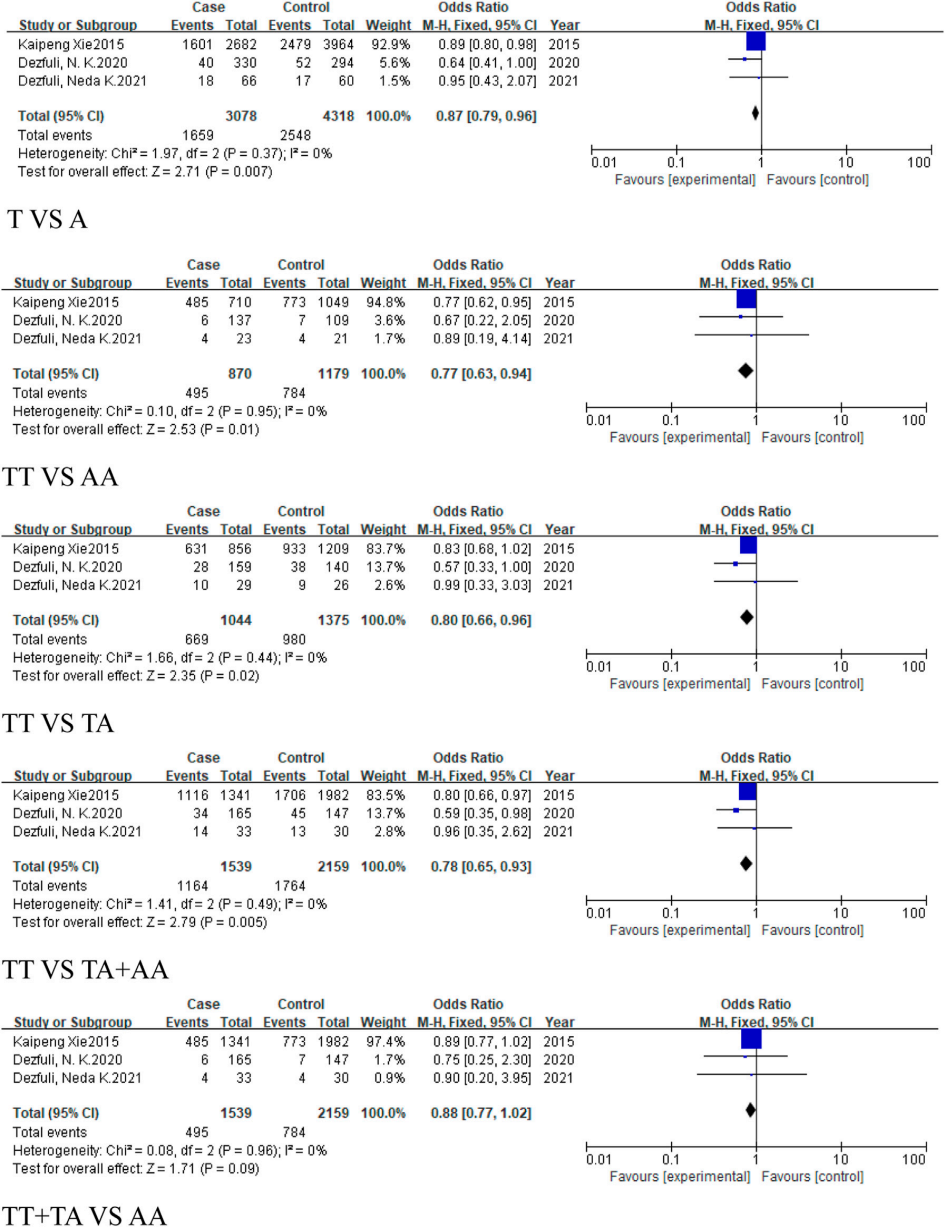
Forest plot of correlation between the microRNA-155 rs767649 (T > A) polymorphism and respiratory system-related cancers.

For the group of respiratory system-related cancers (non-small cell lung cancer), three studies (1,539 cases, 2,159 controls) identified a statistically significant association in the allele model (T vs. A, OR = 0.87, 95%CI [0.79, 0.96], P = 0.007), the homozygous model (TT vs. AA, OR = 0.77, 95%CI [0.63, 0.94], P = 0.01), heterozygous model (TT vs. TA, OR = 0.8, 95%CI [0.66, 0.96], P = 0.02), and the dominant model (TA + AA vs. TT, OR = 0.78, 95%CI [0.65, 0.93], P = 0.005) (Figure 4). No significant association was found in the recessive model.

**FIGURE 5:**
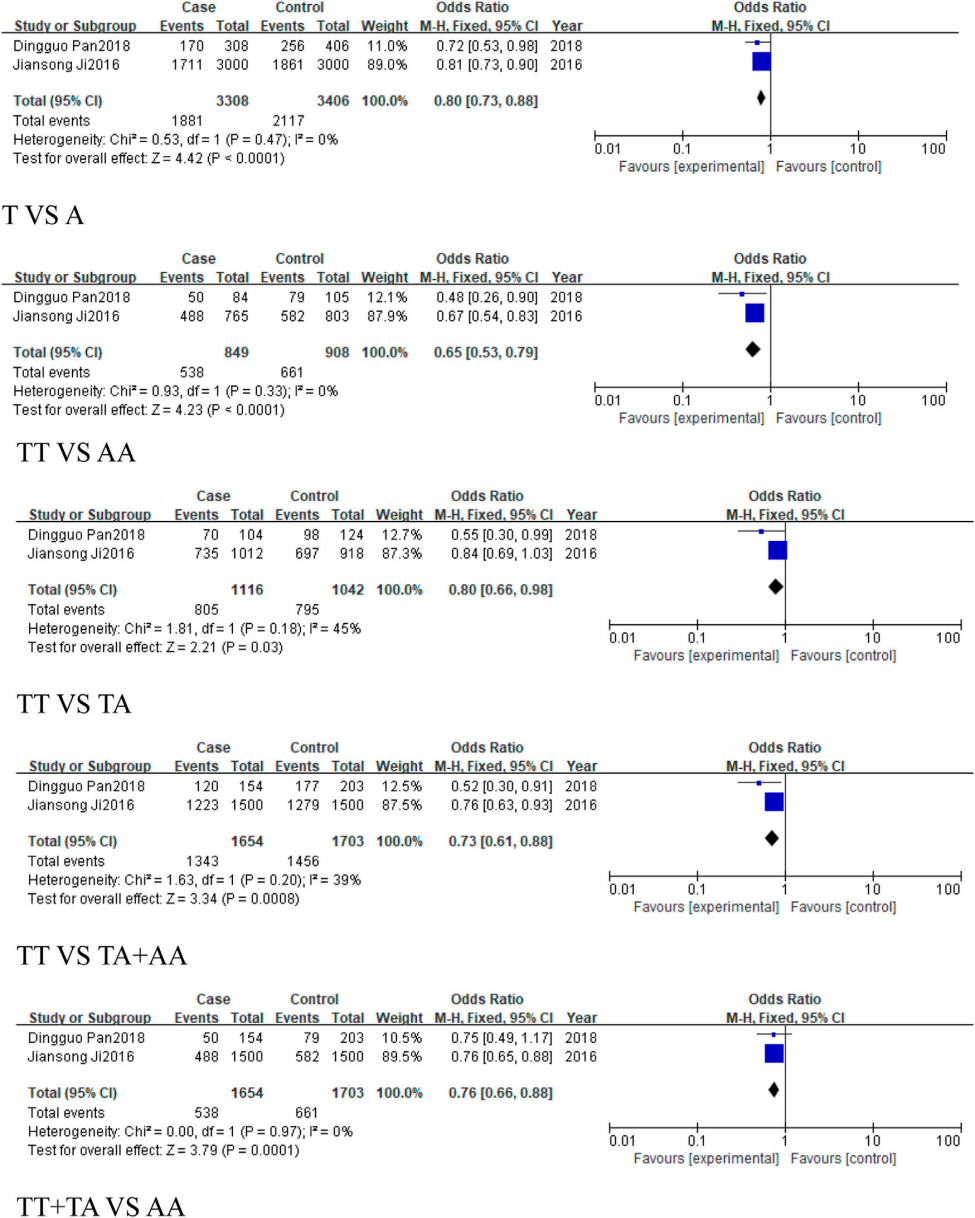
Forest plot of correlation between the microRNA-155 rs767649 (T > A) polymorphism and digestive system-related cancers.

Similarly, 2 studies were included in the subgroup of digestive system-related cancers, including 1,654 cases and 1703 controls. The heterogeneity among the five models was low, and a fixed-effect model was used for analysis. Statistically significant associations were identified in some models for digestive system-related cancers: the allele model (T vs. A, OR = 0.8, 95%CI [0.73, 0.88], P < 0.0001), the homozygous model (TT vs. AA, OR = 0.65, 95%CI [0.53, 0.79], P < 0.0001), heterozygous model (TT vs. TA, OR = 0.8, 95%CI [0.66, 0.98], P = 0.03), the dominant model (TA + AA vs. TT, OR = 0.73, 95%CI [0.61, 0.88], P = 0.0008), and the recessive model (TT + TA vs. AA, OR = 0.76, 95%CI [0.66, 0.88], P = 0.0001) (Figure 5), with significant associations in the allele and homozygous models.

**FIGURE 6:**
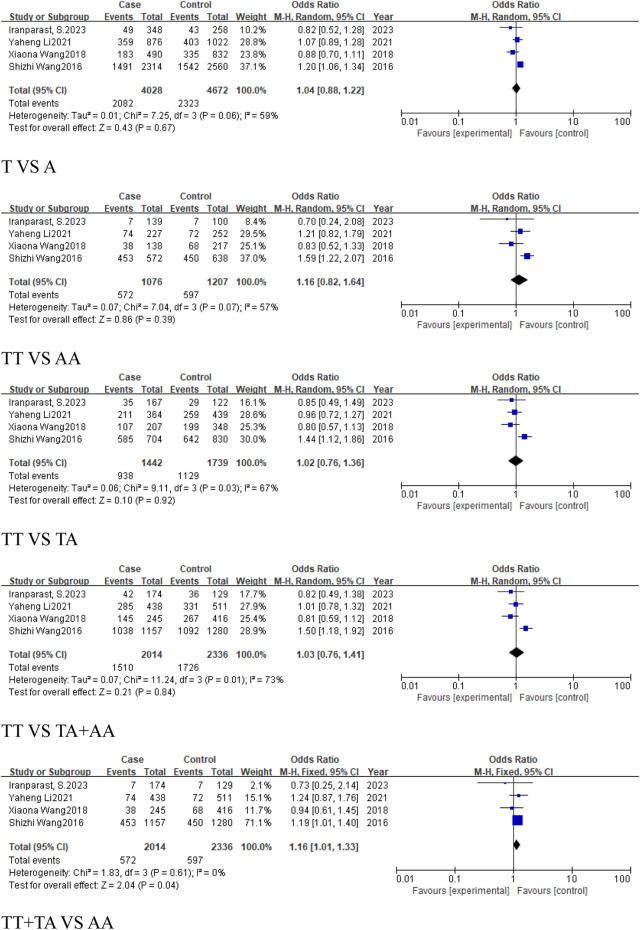
Forest plot of correlation between the microRNA-155 rs767649 (T > A) polymorphism and reproductive system-related cancers.

For reproductive system-related cancers subgroup, 4 studies were included, including 2014 cases and 2,336 controls. The results showed that the rs767649 (T > A) polymorphism increased the risk of reproductive system-related cancers in the recessive model (TT + TA vs. AA, OR = 1.16, 95%CI [1.01, 1.33], P = 0.04) (Figure 6), while other models showed no significant association.

**FIGURE 7:**
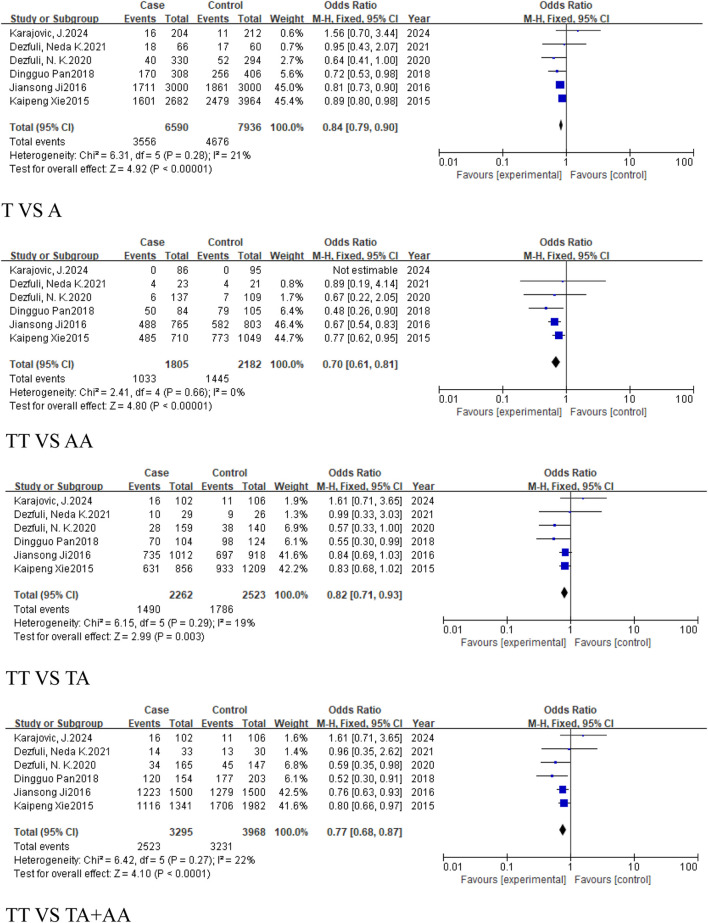
Forest plot of correlation between the microRNA-155 rs767649 (T > A) polymorphism and cancer after exclusion of reproductive system-related cancers.

In the subgroup of cancers, which excludes reproductive system-related cancers, included six studies (3,295 cases, 3,968 controls), all five models showed reduced risk of cancer, including the allele model (T vs. A, OR = 0.84, 95%CI [0.79, 0.90], P < 0.00001), homozygous model (TT vs. AA, OR = 0.7, 95%CI [0.61, 0.81], P < 0.00001), heterozygous model (TT vs. TA, OR = 0.82, 95%CI [0.71, 0.93], P = 0.003), dominant model (TA + AA vs. TT, OR = 0.77, 95%CI [0.68, 0.87], P < 0.0001), and recessive model (TT + TA vs. AA, OR = 0.82, 95%CI [0.74, 0.91], P = 0.0001) (Figure 7).

#### 3.2.3 The analysis of the correlation between the *MIR155HG* rs767649 (T > A) polymorphism and cancer in different regions and races

This analysis aimed to investigate the impact of geographical variation on the association between the rs767649 (T>A) polymorphism and cancer risk. Subgroup analyses were conducted for Asian, European, East Asian, and Caucasian populations.

**FIGURE 8:**
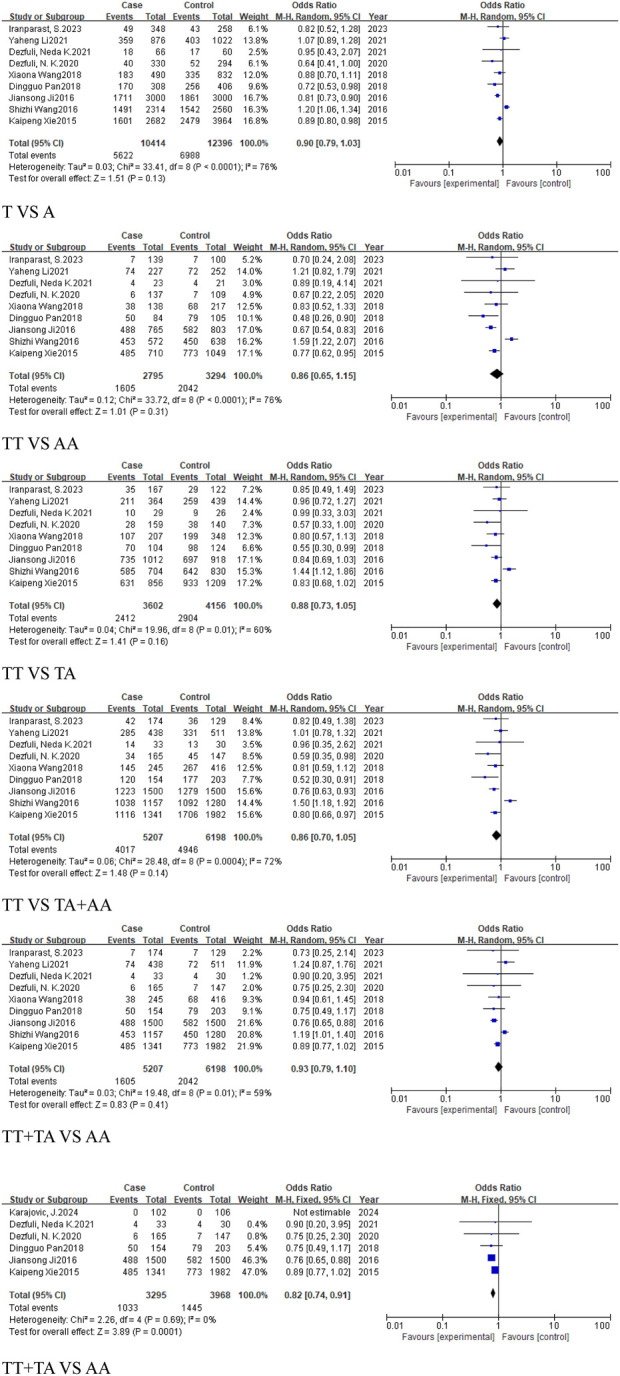
Forest plot of correlation between the microRNA-155 rs767649 (T > A) polymorphism and cancer (Asian subgroup).

The meta-analysis of the regional subgroups included a total of 10 studies comprising 5,309 cases and 6,304 controls. Based on geographical differences, the studies were categorized into Asian and European subgroups (Table 2). The Asian subgroup comprised 9 studies, (5,207 cases, 6,198 controls), and no statistically significant associations were observed across any genetic models, including the allele model (T vs. A, OR = 0.9, 95% CI [0.79, 1.03]), homozygous model (TT vs. AA, OR = 0.86, 95% CI [0.65, 1.15]), heterozygous model (TT vs. TA, OR = 0.88, 95% CI [0.73, 1.05]), dominant model (TA + AA vs. TT, OR = 0.86, 95% CI [0.70, 1.05]), and recessive model (TT + TA vs. AA, OR = 0.93, 95%CI [0.79, 1.10]) (Figure 8).

**FIGURE 9:**
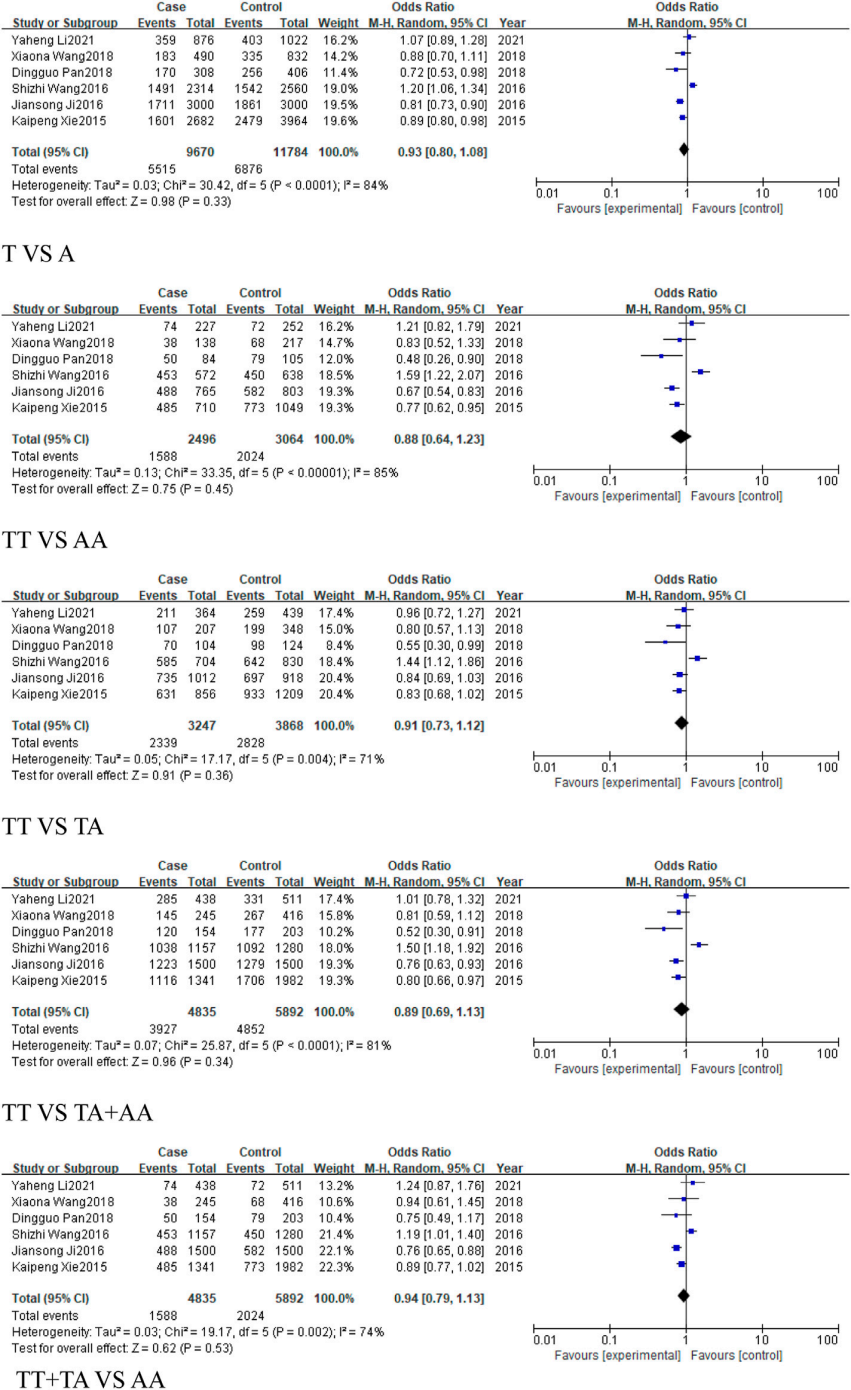
Forest plot of correlation between the microRNA-155 rs767649 (T > A) polymorphism and cancer (East Asian subgroup).

**FIGURE 10:**
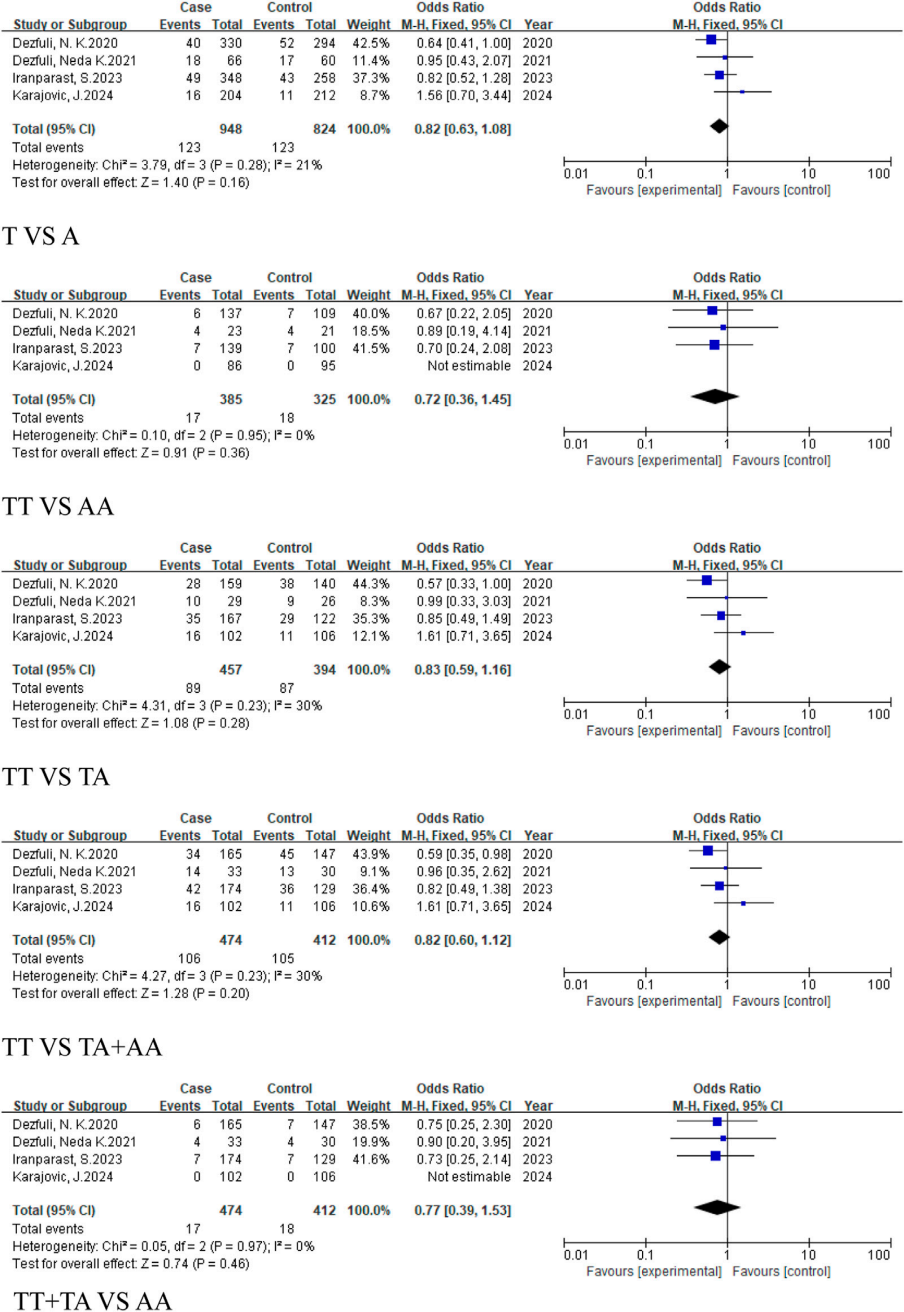
Forest plot of correlation between the microRNA-155 rs767649 (T > A) polymorphism and cancer (Caucasian subgroup).

The European subgroup consisted of only one study, and no pooled analysis was conducted due to insufficient data (Table 4). The East Asian subgroup included 6 studies (4,835 cases, 5,892 controls), with results showing no statistically significant associations across all genetic models: allele model (T vs. A, OR = 0.93, 95% CI [0.80, 1.08]), homozygous model (TT vs. AA, OR = 0.88, 95% CI [0.64, 1.23]), heterozygous model (TT vs. TA, OR = 0.91, 95% CI [0.73, 1.12]), dominant model (TA + AA vs. TT, OR = 0.89, 95% CI [0.69, 1.13]), and recessive model (TT + TA vs. AA, OR = 0.94, 95%CI [0.79, 1.13]) (Figure 9). For the Caucasian subgroup, which consisted of 4 studies with 474 cases and 412 controls, the rs767649 polymorphism showed no statistically significant associations with cancer risk across all five genetic models: allelic model (T vs. A, OR = 0.82, 95% CI [0.63, 1.08]), homozygous model (TT vs. AA, OR = 0.72, 95% CI [0.36, 1.45]), heterozygous model (TT vs. TA, OR = 0.83, 95% CI [0.59, 1.16]), dominant model (TA + AA vs. TT, OR = 0.82, 95% CI [0.60, 1.12]), and recessive model (TT + TA vs. AA, OR = 0.77, 95% CI [0.39, 1.53]) (Figure 10).

### 3.3 The analysis of *MIR155HG* rs928883 (G > A) polymorphism

The meta-analysis of rs928883 (G > A) polymorphism comprised a total of 4 studies, with 2,176 cases and 1957 controls. The heterogeneity test of this study was evaluated using the I^2^ test, the results indicated that the included studies exhibited high heterogeneity, except for the recessive model. Therefore, a random-effects model was used to combine the research results in four genetic models. The meta-analysis results of the association between rs928883 (G > A) polymorphism and the risk of cancer and its subgroups are shown in the table (Table 5).

**TABLE 5:** The meta-analysis results of microRNA-155 rs928883 (G > A) polymorphism and its correlation with the risk of cancer and its subgroups.

	Type	OR (95%)	95% CI	z	p	Test of heterogeneity		Analysis model
						I2	P*	
Cancer (4)	G vs. A	1.01	[0.81, 1.27]	0.11	0.91	81	0.001	Random-effects model
	GG vs. AA	1.12	[0.63, 2.01]	0.39	0.7	83	0.0004	Random-effects model
	GG vs. GA	1.06	[0.77, 1.45]	0.34	0.74	78	0.004	Random-effects model
	GA + AA vs. GG	1.04	[0.75, 1.45]	0.26	0.8	82	0.001	Random-effects model
	GG + GAvs AA	0.96	[0.65, 1.43]	0.18	0.86	74	0.009	Random-effects model
Carcinoma (3)	G vs. A	0.93	[0.75, 1.16]	0.62	0.53	77	0.01	Random-effects model
	GG vs. AA	0.86	[0.57, 1.31]	0.7	0.48	74	0.02	Random-effects model
	GG vs. GA	1.06	[0.67, 1.67]	0.23	0.82	85	0.001	Random-effects model
	GA + AA vs. GG	1	[0.63, 1.56]	0.02	0.98	86	0.0008	Random-effects model
	GG + GAvs AA	0.82	[0.69, 0.99]	2.11	0.03	0	0.64	Fixed-effects model
Digestive system cancers (3)	G vs. A	0.93	[0.75, 1.16]	0.62	0.53	77	0.01	Random-effects model
	GG vs. AA	0.86	[0.57, 1.31]	0.7	0.48	74	0.02	Random-effects model
	GG vs. GA	1.06	[0.67, 1.67]	0.23	0.82	85	0.001	Random-effects model
	GA + AA vs. GG	1	[0.63, 1.56]	0.02	0.98	86	0.0008	Random-effects model
	GG + GAvs AA	0.82	[0.69, 0.99]	2.11	0.03	0	0.64	Fixed-effects model
Asian (3)	G vs. A	0.93	[0.75, 1.16]	0.62	0.53	77	0.01	Random-effects model
	GG vs. AA	0.86	[0.57, 1.31]	0.7	0.48	74	0.02	Random-effects model
	GG vs. GA	1.06	[0.67, 1.67]	0.23	0.82	85	0.001	Random-effects model
	GA + AA vs. GG	1	[0.63, 1.56]	0.02	0.98	86	0.0008	Random-effects model
	GG + GAvs AA	0.82	[0.69, 0.99]	2.11	0.03	0	0.64	Fixed-effects model
East Asian (3)	G vs. A	0.93	[0.75, 1.16]	0.62	0.53	77	0.01	Random-effects model
	GG vs. AA	0.86	[0.57, 1.31]	0.7	0.48	74	0.02	Random-effects model
	GG vs. GA	1.06	[0.67, 1.67]	0.23	0.82	85	0.001	Random-effects model
	GA + AA vs. GG	1	[0.63, 1.56]	0.02	0.98	86	0.0008	Random-effects model
	GG + GAvs AA	0.82	[0.69, 0.99]	2.11	0.03	0	0.64	Fixed-effects model

#### 3.3.1 The analysis of *MIR155HG* rs928883 (G > A) polymorphism in overall cancer

**FIGURE 11:**
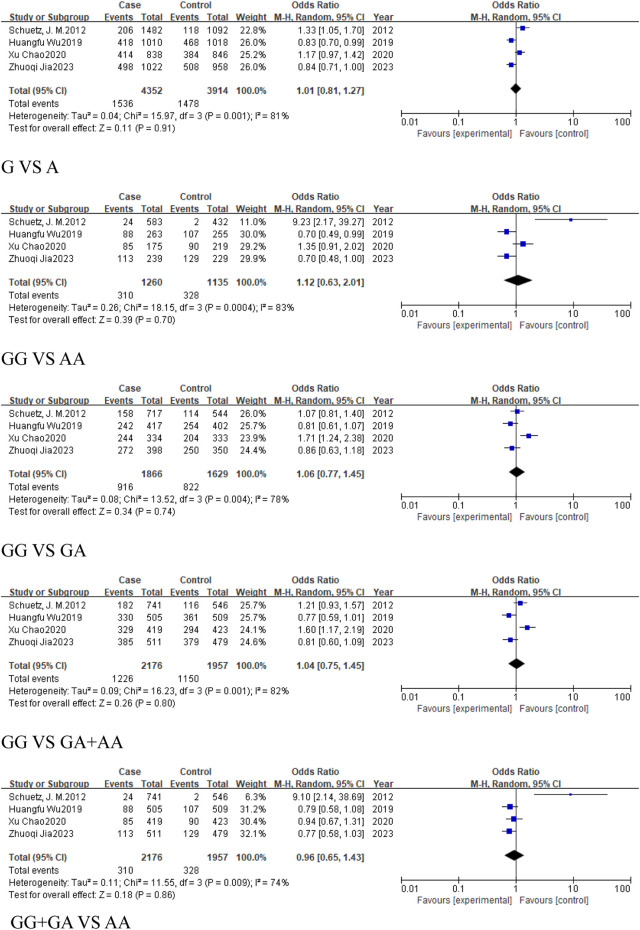
Forest plot of correlation between the microRNA-155 rs928883 (G > A) polymorphism and cancer.

Four studies, comprising 2,176 cancer cases and 1,957 controls, were included in the cancer group. The results revealed no statistically significant association across all genetic models: the allele model (G vs. A, OR = 1.01, 95% CI [0.81, 1.27]), homozygous model (GG vs. AA, OR = 1.12, 95% CI [0.63, 2.01]), heterozygous model (GG vs. GA, OR = 1.06, 95% CI [0.77, 1.45]), dominant model (GA + AA vs. GG, OR = 1.04, 95% CI [0.75, 1.45]), and the recessive model (GG + GA vs. AA, OR = 0.96, 95% CI [0.65, 1.43]) (Figure 11). This suggests that there is no significant association between rs928883 (G > A) polymorphism and the risk of cancer in the overall cancer analysis (Table 5).

#### 3.3.2 The analysis of *MIR155HG* rs928883 (G > A) polymorphism in cancer subgroups

**FIGURE 12:**
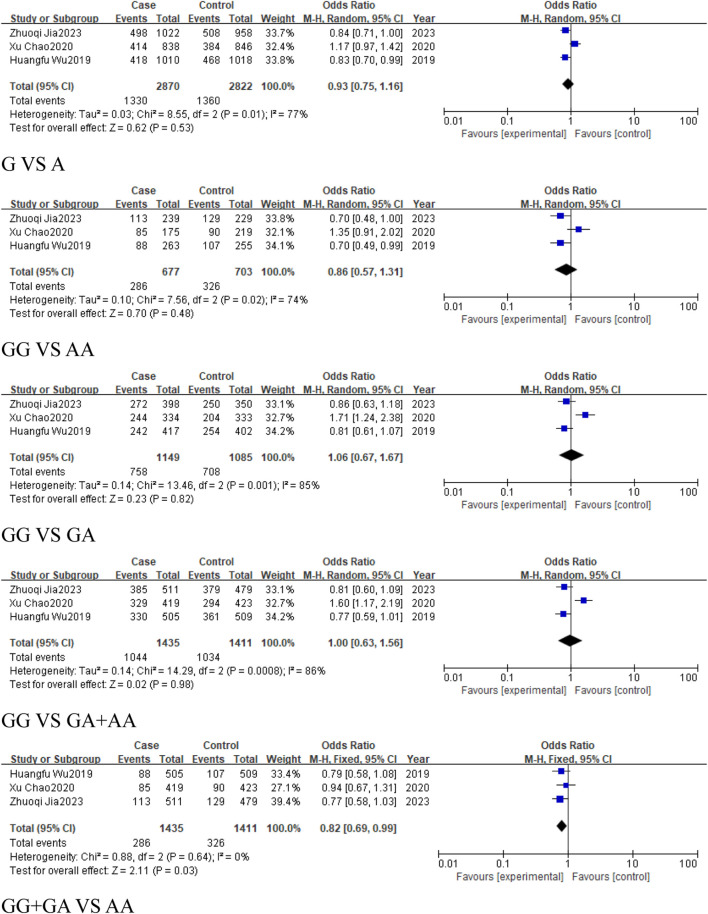
Forest plot of correlation between the microRNA-155 rs928883 (G > A) polymorphism and digestive system-related cancers.

Three studies, comprising 1,435 cases and 1,411 controls, were included in the subgroup of digestive system-related cancers. The results indicated a statistically significant association in the recessive model (GG + GA vs. AA, OR = 0.82, 95%CI [0.69, 0.99], P = 0.03), suggesting that the rs928883 (G > A) polymorphism may play a role in reducing the risk of digestive system-related cancers (Figure 12). Similarly, in the analysis of epithelial-origin malignancies and the subgroup of Asian and East Asian populations, the rs928883 (G > A) polymorphism was found to be associated with cancer only in the recessive model (GG + GA vs. AA, OR = 0.82, 95%CI [0.69, 0.99], P = 0.03), which was statistically significant as a protective factor (Figure 12).

### 3.4 The analysis of *MIR155HG* rs1893650 (T > C) polymorphism

**FIGURE 13:**
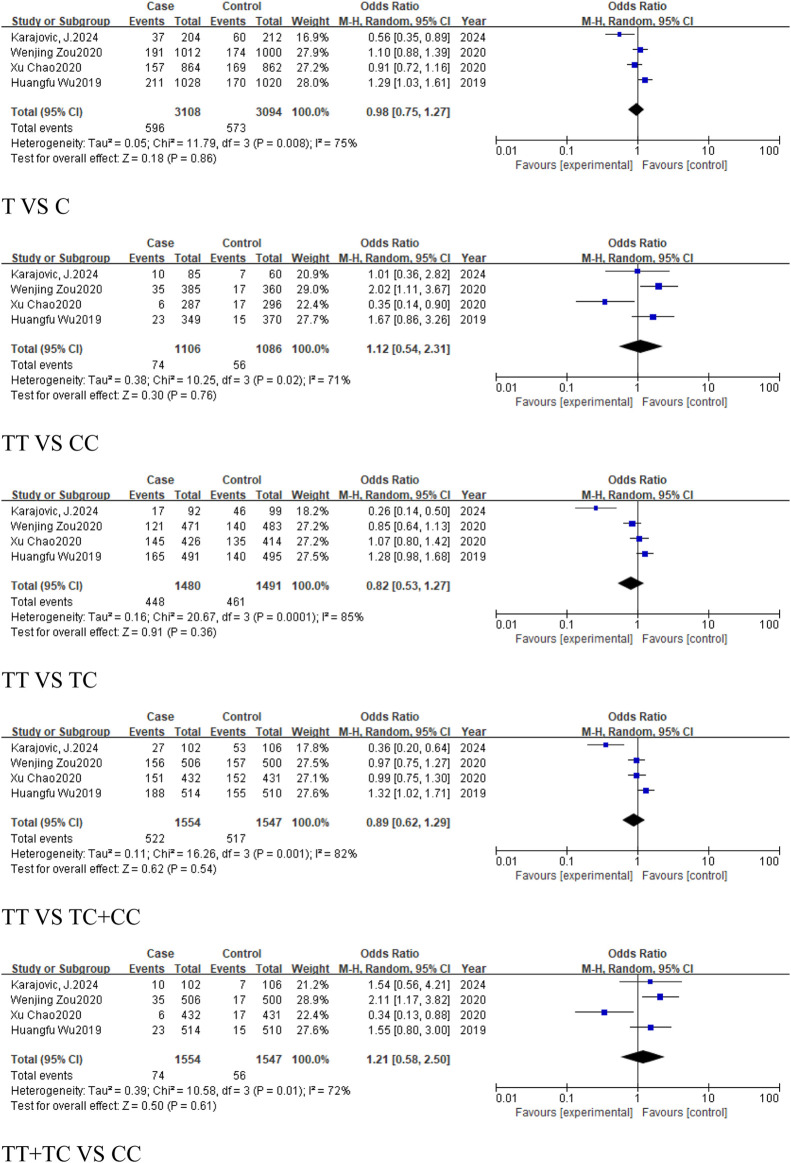
Forest plot of correlation between the microRNA-155 rs1893650 (T > C) polymorphism and cancers.

**FIGURE 14:**
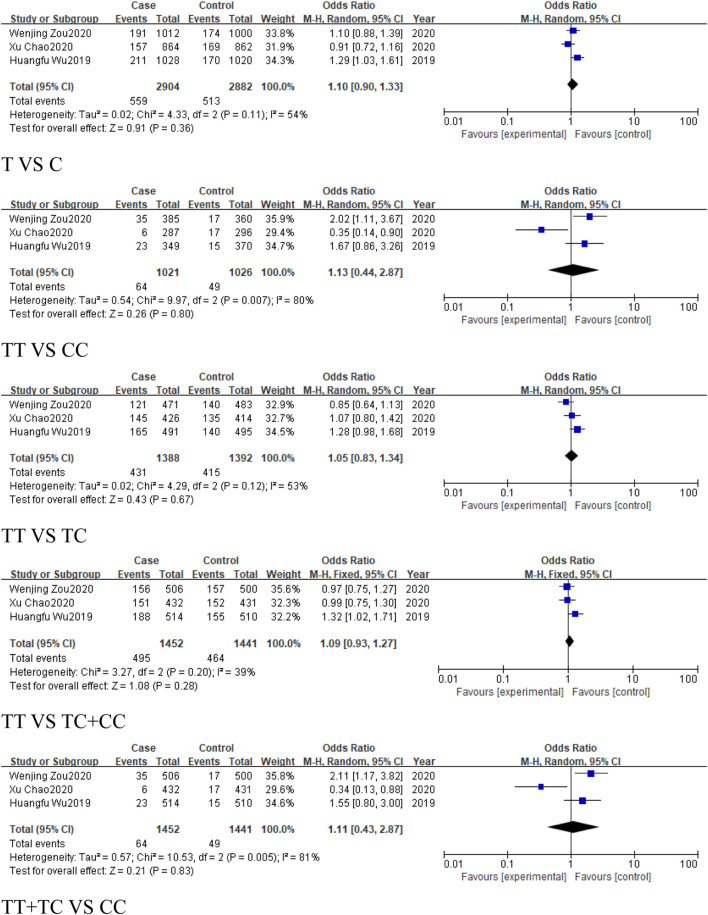
Forest plot of correlation between the microRNA-155 rs1893650 (T > C) polymorphism and digestive system-related cancers.

A total of 4 studies were included in the group of cancer-related studies, involving 1,554 cancer cases and 1,547 controls. The included studies, except for the dominant model, exhibited high heterogeneity, and therefore a random-effects model was used for the analysis (Table 6). The meta-analysis results showed no association between rs1893650 (T > C) and cancer in any of the five genetic models (Figure 13). Similarly, in subgroup analyses for digestive system-related cancers, as well as for Asian and East Asian populations, no significant association was found between rs1893650 and cancer risk in any of the five genetic models (Figure 14). These findings suggest that the rs1893650 (T > C) polymorphism is not significantly associated with cancer risk or the risk in its subgroups. Given the limited number of included studies, the meta-analysis results should be interpreted with caution.

**TABLE 6:** The meta-analysis results of microRNA-155 rs1893650 (T > C) polymorphism and its correlation with the risk of cancer and its subgroups.

	Type	OR (95%)	95% CI	z	p	Test of heterogeneity		Analysis model
						I2	P*	
Cancer (4)	T vs. C	0.98	[0.75, 1.27]	0.18	0.86	75	0.008	Random-effects model
	TT vs. CC	1.12	[0.54, 2.31]	0.3	0.76	71	0.02	Random-effects model
	TT vs. TC	0.82	[0.53, 1.27]	0.91	0.36	85	0.0001	Random-effects model
	TC + CC vs. TT	0.89	[0.62, 1.29]	0.62	0.54	82	0.001	Random-effects model
	TT + TC vs. CC	1.21	[0.58, 2.50]	0.5	0.61	72	0.01	Random-effects model
Digestive system cancers (3)	T vs. C	1.1	[0.90, 1.33]	0.91	0.36	54	0.11	Random-effects model
	TT vs. CC	1.13	[0.44, 2.87]	0.26	0.8	80	0.007	Random-effects model
	TT vs. TC	1.05	[0.83, 1.34]	0.43	0.67	53	0.12	Random-effects model
	TC + CC vs. TT	1.09	[0.93, 1.27]	1.08	0.28	39	0.2	Fixed-effects model
	TT + TC vs. CC	1.11	[0.43, 2.87]	0.21	0.83	81	0.005	Random-effects model
Asian (3)	T vs. C	1.1	[0.90, 1.33]	0.91	0.36	54	0.11	Random-effects model
	TT vs. CC	1.13	[0.44, 2.87]	0.26	0.8	80	0.007	Random-effects model
	TT vs. TC	1.05	[0.83, 1.34]	0.43	0.67	53	0.12	Random-effects model
	TC + CC vs. TT	1.09	[0.93, 1.27]	1.08	0.28	39	0.2	Fixed-effects model
	TT + TC vs. CC	1.11	[0.43, 2.87]	0.21	0.83	81	0.005	Random-effects model
East Asian (3)	T vs. C	1.1	[0.90, 1.33]	0.91	0.36	54	0.11	Random-effects model
	TT vs. CC	1.13	[0.44, 2.87]	0.26	0.8	80	0.007	Random-effects model
	TT vs. TC	1.05	[0.83, 1.34]	0.43	0.67	53	0.12	Random-effects model
	TC + CC vs. TT	1.09	[0.93, 1.27]	1.08	0.28	39	0.2	Fixed-effects model
	TT + TC vs. CC	1.11	[0.43, 2.87]	0.21	0.83	81	0.005	Random-effects model

#### 3.4.1 Heterogeneity testing and publication bias

Due to the limited number of original articles included in this research, as well as the scarcity of studies for rs767649 (T > A), rs928883 (G > A), and rs1893650 (T > C) with no more than 10 studies respectively, we did not evaluate the presence of publication bias (Sterne et al., 2001). Sensitivity analysis was not conducted for studies with low heterogeneity. However, for studies with high heterogeneity, further subgroup analysis is performed based on the existing subtyping to explore potential sources of heterogeneity.

## 5 Discussion

As a significant miRNA, miR-155 was first discovered in 2002 (Lagos-Quintana et al., 2002). Since then, miR-155 has been shown to play a crucial role in various biological processes, including immune regulation, inflammatory responses, and cell proliferation. It regulates the expression of target genes, participating in multiple cellular processes such as proliferation, differentiation, and apoptosis, making it a research focus in fields such as immune diseases and cardiovascular diseases. In recent years, studies have found that miR-155 is an evolutionarily conserved miRNA regulated by the activator protein-1 and nuclear factor-κB complexes. By binding to multiple target genes, miR-155 is involved in the development and progression of cancer (Faraoni et al., 2009; Thai et al., 2007). SNPs may affect gene transcription and/or protein expression, thereby influencing individual susceptibility to diseases. Research suggests that SNPs in the miR-155 gene are closely associated with various malignant tumors (Iranparast et al., 2023; Karajovic et al., 2024; Xie et al., 2015). To the best of our knowledge, rs767649 (T > A), rs928883 (G > A), and rs1893650 (T > C) are the three most studied SNP sites in the *MIR155HG*. This study represents the first meta-analysis that investigates the correlation between the *MIR155HG* polymorphism and the susceptibility to cancer and its subgroups. We summarize and analyze the potential impact of mutations at these specific sites on the development of cancer. By systematically reviewing the existing research in this field, this study aims to provide valuable insights and serve as a reference for future clinical investigations in this area.

This meta-analysis, encompassing data from 10 published articles (5,309 cancer cases and 6,304 controls), investigated the association between the rs767649 (T > A) polymorphism and cancer. There is no significant correlation between rs767649 polymorphism and the susceptibility to cancer.

Notably, the aforementioned studies generally exhibit high heterogeneity in genetic models, which may be attributed to differences in the types of diseases chosen by various studies. Different tumor types may have distinct pathogenesis mechanisms, potentially leading to different roles of *MIR155HG*. To address this possibility, our study further explored the association between the rs767649 (T > A) polymorphism and cancer subgroups, analyzing the correlation of rs767649 with cancers of the respiratory system, digestive system, and reproductive system. When stratified by cancer type, this significant heterogeneity was reduced or disappeared. Three original studies analyzed the association between the rs767649 (T > A) polymorphism and respiratory cancer risk, including 1539 NSCLC cases and 2,159 healthy controls. The analysis showed that the rs767649 polymorphism reduced the risk of NSCLC across four genetic models: allele model, homozygous model, heterozygous model, and dominant model. Compared with the TT genotype, the AA genotype reduced the risk of NSCLC by 23% (P = 0.01), and the TA genotype reduced it by 20% (P = 0.02). The TA + AA genotypes reduced the OR for NSCLC by 22% (P = 0.005), suggesting that the A allele may be a key factor influencing NSCLC risk. Our study also analyzed the association between the rs767649 (T>A) polymorphism and the risk of digestive system-related cancers using data from two reports. Meta-analysis results showed that this SNP was associated with a reduced risk of digestive system cancers across all five genetic models, with statistically significant differences in the allele model, homozygous model, dominant model, and recessive model. These findings suggest that tumor type and sample size play important roles in the study of SNP-cancer associations. Additionally, we examined the association between the rs767649 (T>A) polymorphism and the risk of reproductive system-related cancers using data from four original studies. The results showed that all five genetic models increased the risk of reproductive system cancers, consistent with the findings of Wang et al. (Wang et al., 2016), but this correlation only reached statistical significance in the recessive model (P = 0.04). Given the relatively small sample size in this cancer type, these results should be interpreted with caution. Research has shown that factors such as hormones, lifestyle, and environmental factors lead to differences in cancer incidence between genders (Borg et al., 2024; Fu et al., 2021; Zheng et al., 2017). To minimize the influence of gender on the study results, we excluded original studies related to reproductive system cancers, such as cervical and breast cancers. In the cancer subgroups excluding reproductive system cancers, we found that the rs767649 (T > A) polymorphism reduced cancer risk across all five genetic models (allele model, homozygous model, heterozygous model, dominant model, and recessive model), with statistically significant differences observed in all five models.

The study populations selected in different studies vary, and these populations may possess distinct genetic backgrounds. The role of SNPs at these sites may be influenced by different genetic backgrounds, which could affect the heterogeneity of the genetic models in this study. Research has reported an association between rs767649 (T > A) polymorphism and cancer risk in East Asian populations, while such associations have been less frequently reported in Caucasian populations. A subgroup analysis based on the geographic origin of the samples revealed no statistically significant association between the two in the Asian group. Regarding the heterogeneity in regional groups, we reviewed some details of the included studies, focusing on the racial characteristics of the populations. The populations included both East Asians and Caucasians, which could be a source of heterogeneity. When conducting a subgroup analysis based on race, the heterogeneity in the Caucasian subgroup disappeared, no correlation was found between rs767649 (T>A) polymorphism and cancer risk. This suggests that the relationship between this SNP and cancer risk remains unclear across different regions and ethnicities.

The meta-analysis of this study shows that the rs928883 (G > A) polymorphism is not significantly associated with cancer risk in the overall cancer analysis. When classified according to tumor types, it was found that the recessive model of rs928883 (G > A) reduces the risk of digestive system-related cancers and serves as a protective factor against the occurrence of such cancers. Following this, we conducted analyses by region and ethnic subgroups, finding that in the Asian subgroup and the East Asian ethnic subgroup, the recessive model of rs928883 (G > A) polymorphism is significantly associated with cancer susceptibility. It is noteworthy that the recessive model of this SNP acts as a protective factor in cancer occurrence. The original studies in the European subgroup and Caucasian ethnic subgroup included in this study consisted of only one paper (Schuetz et al., 2012), and we did not perform a meta-analysis on the association between rs928883 polymorphism and cancer risk in this subgroup. However, Schuetz et al. were the first to report the association between rs928883 polymorphism and marginal zone lymphoma.

In existing studies, there is limited original research on the relationship between the rs1893650 (T > C) polymorphism located in the intron region of *MIR155HG* and cancer risk. Published cancer-related studies indicate an association between rs1893650 (T > C) polymorphism and cancer susceptibility. However, its role varies across different cancer types. Karajovic et al. found that rs1893650 is negatively correlated with thyroid cancer susceptibility, suggesting that rs1893650 could serve as a biomarker for indicating potential risk and prognosis of thyroid cancer (Karajovic et al., 2024). In contrast, Zou et al. reported that the *MIR155HG* rs1893650 (T > C) polymorphism increases the risk of gastric cancer (Zou et al., 2020). The discrepancy between these findings may be due to the different types of diseases studied, as the mechanisms of different tumor types may vary, leading to different roles for *MIR155HG*. The results of the meta-analysis in this study do not support the association between rs1893650 polymorphism and cancer or its subtypes. Furthermore, most current studies on the rs1893650 polymorphism focus on its association with the risk of cardiovascular diseases (heart disease, atherosclerosis), metabolic diseases (obesity, diabetes), and autoimmune diseases (rheumatoid arthritis, systemic lupus erythematosus).

Our research has certain limitations. Firstly, the number of studies included in our analysis is limited, which may lead to false positive or false negative results, particularly in terms of sample size for each subgroup. This limitation hinders a comprehensive exploration of the relationship between low-frequency variations and the risk of cancer. Secondly, the control group for the primary studies used in our study is derived from hospitals, which may not accurately represent the general population. It is challenging to completely eliminate selection bias. Finally, we extracted basic information from each study, such as the country of the study subjects, their race, and genetic mutation sites. However, we still cannot avoid the influence of some confounding factors, such as age, familial relationships, smoking, and drug history. These limitations may potentially reduce the accuracy of the final results. Given these limitations, the results of our study should be carefully interpreted and further elaborated upon with a larger sample size in a systematic manner.

## 6 Conclusion

This study is the first meta-analysis to systematically examine the associations between *MIR155HG* polymorphisms and cancer susceptibility. We found that rs767649 (T > A) is associated with reduced risk in NSCLC and digestive system cancers but increased risk in reproductive system cancers, highlighting its tumor-specific effects. Excluding reproductive system cancers further confirmed its protective role in non-reproductive cancers. Similarly, rs928883 (G > A) showed a protective effect in digestive system cancers, particularly in East Asian populations, while no significant associations were observed for rs1893650 (T > C). Our findings provide novel insights into the roles of *MIR155HG* polymorphisms as potential biomarkers for cancer risk stratification. These results underscore their utility in precision oncology for developing targeted screening and prevention strategies, warranting further validation through large-scale studies.

## Data Availability

The original contributions presented in the study are included in the article/Supplementary Material, further inquiries can be directed to the corresponding authors.
