# Supplementary material for: The relationship of miR-155 host gene polymorphism in the susceptibility of cancer: a systematic review and meta-analysis
Source: Front Genet. 2025 Mar 6;16:1517513. doi: 10.3389/fgene.2025.1517513 (PMC11922843; doi:10.3389/fgene.2025.1517513)
Supplement: Supplementary file 1 [file Table1.docx]

**PubMed**

((("Polymorphism, Single Nucleotide"[Mesh]) OR ((((((((single nucleotide polymorphism[Title/Abstract]) OR (Single Nucleotide Polymorphisms[Title/Abstract])) OR (SNPs[Title/Abstract])) OR (SNP[Title/Abstract])) OR (polymorphism[Title/Abstract])) OR (Variation[Title/Abstract])) OR (variant[Title/Abstract])) OR (mutation[Title/Abstract]))) OR (((((genetic polymorphism[Title/Abstract]) OR (Genetic Polymorphisms[Title/Abstract])) OR (Gene Polymorphism[Title/Abstract])) OR (Gene Polymorphisms[Title/Abstract])) OR ("Polymorphism, Genetic"[Mesh]))) AND (((((((((((miR-155[Title/Abstract]) OR (miR155[Title/Abstract])) OR (hsa-mir-155 microRNA[Title/Abstract])) OR (miRNA-155[Title/Abstract])) OR (microRNA-155[Title/Abstract])) OR (miR-155 microRNA[Title/Abstract])) OR (miR-155-5p[Title/Abstract])) OR (MIR155HG[Title/Abstract])) OR (MicroRNA 155 Host Gene[Title/Abstract])) OR (miR-155 Host Gene[Title/Abstract])) OR ("MIRN155 microRNA, human" [Supplementary Concept]))

Web of Science

“miR-155” or “miR155” or “hsa-mir-155 microRNA” or “miRNA-155” or “microRNA-155” or “miR-155 microRNA” or “miR-155-5p” or “MIR155HG” or “MicroRNA 155 Host Gene” or “miR-155 Host Gene”

“Genetic polymorphism” or “Genetic Polymorphisms” or “Gene Polymorphism” or “Gene Polymorphisms” or “single nucleotide polymorphism” or “Single Nucleotide Polymorphisms” or “SNPs” or “SNP” or “polymorphism” or “Variation” or “variant” or “mutation”

知网

(“miR-155” or “miR155” or “hsa-mir-155 microRNA” or “miRNA-155” or “microRNA-155” or “miR-155 microRNA” or “miR-155-5p” or “MIR155HG” or “MicroRNA 155 Host Gene” or “miR-155 Host Gene”) and ("基因多态性" OR "遗传多态性" OR "基因变异" OR "SNP" OR "单核苷酸多态性")
